# Annual Research Review: Educational neuroscience: progress and prospects

**DOI:** 10.1111/jcpp.12973

**Published:** 2018-10-22

**Authors:** Michael S. C. Thomas, Daniel Ansari, Victoria C. P. Knowland

**Affiliations:** ^1^ Centre for Educational Neuroscience Department of Psychological Science Birkbeck University of London London UK; ^2^ Department of Psychology & Faculty of Education Western University London ON Canada; ^3^ Department of Psychology University of York York UK

**Keywords:** Educational neuroscience, translation, intervention, policy, neuromyths

## Abstract

Educational neuroscience is an interdisciplinary research field that seeks to translate research findings on neural mechanisms of learning to educational practice and policy and to understand the effects of education on the brain. Neuroscience and education can interact directly, by virtue of considering the brain as a biological organ that needs to be in the optimal condition to learn (‘brain health’); or indirectly, as neuroscience shapes psychological theory and psychology influences education. In this article, we trace the origins of educational neuroscience, its main areas of research activity and the principal challenges it faces as a translational field. We consider how a pure psychology approach that ignores neuroscience is at risk of being misleading for educators. We address the major criticisms of the field comprising, respectively, a priori arguments against the relevance of neuroscience to education, reservations with the current practical operation of the field, and doubts about the viability of neuroscience methods for diagnosing disorders or predicting individual differences. We consider future prospects of the field and ethical issues it raises. Finally, we discuss the challenge of responding to the (welcome) desire of education policymakers to include neuroscience evidence in their policymaking, while ensuring recommendations do not exceed the limitations of current basic science.

## Abstract 2

Read the Commentary on this article at doi: 10.1111/jcpp.13030

## Introduction

Educational neuroscience is an interdisciplinary research field that seeks to translate research findings on neural mechanisms of learning to educational practice and policy. There are equivalent fields that seek to translate neuroscience findings to law (e.g. Royal Society, 2011a), economics (e.g. Glimcher & Fehr, 2013) and social policy (e.g. Royal Society, 2011b), drawing on research in behavioural regulation, decision‐making, reward, empathy and moral reasoning. The field is also a basic science that studies how education changes the brain, and what the mechanisms are that lead to behavioural change (or the absences thereof) through education. The relevance of neurobiology to education was recognised throughout the 20th century (e.g. Thorndike, 1926), but it was not until the 1990s and the “Decade of the Brain” (Jones & Mendell, 1999) that technological advances in in vivo imaging of brain function led to the theoretical advances that made educational neuroscience viable as a field (Varma, McCandliss, & Schwartz, 2008).

Despite strong critics (Bishop, 2014; Bowers, 2016a; Bruer, 1997) and vigorous ongoing debate about the merits of bringing knowledge from neuroscientific research to bear on educational problems (Gabrieli, 2016; Howard‐Jones et al., 2016), the potential connections between neuroscience and education are being actively explored across the globe. Different labels have been used to describe such efforts, such as *Neuroeducation*,* Educational Neuroscience* and *Mind, Brain and Education*. The growth of the field has led to the establishment of new societies and groups: the International Mind, Brain and Education Society (IMBES; www.imbes.org) was founded in 2004; in 2009, the European Association for Research on Learning and Instruction (EARLI) founded a Special Interest Group called ‘Neuroscience and Education’ which has been holding biannual meetings since 2010. New journals have been established, such as ‘Trends in Neuroscience and Education’, ‘Mind, Brain and Education and ‘Educational Neuroscience’, which attract theoretical and empirical work that explores the intersections of neuroscience, psychology and education. There has been a growth in postgraduate courses in educational neuroscience at leading international universities such as Harvard and the Universities of London and Bristol. Moreover, there have been reports commissioned by high profile organisations, such as the Royal Society (“Neuroscience: implications for education and lifelong learning”; Royal Society, 2011c) and the Organization for Economic Cooperation and Development (“Understanding the Brain: The Birth of a Learning Science”; OECD, 2007).

Nevertheless, translation from neuroscience research to education is difficult. As Howard‐Jones (2010, p. xi) comments, no classroom‐ready knowledge from neuroscience is ever likely to exist. Translation is an extended process and it begins with a foundation of basic science. The complexity of learning in the brain and the state of current scientific knowledge mean that there is a risk of premature translation before the foundation is established. The risk is heightened by the legitimate desire of policymakers to use scientific evidence to inform their education policies (e.g. Willetts, 2018), the enthusiasm that educators have to inform their teaching with insights into how the brain works, and the desire of commercial companies to sell new techniques to schools using the latest neuroscience findings as window dressing (Brookman‐Byrne & Thomas, 2018; Goswami, 2006; Howard‐Jones, 2014a). Moreover, the interaction of the disciplines of neuroscience, psychology and education has sometimes been characterised by competition rather than collaboration, and education researchers remain suspicious of the hype surrounding educational neuroscience. Two recent comments from leading education researchers illustrate these views: “There seem to be three times as many articles considering the *promise* of neuroscience being brought to bear on education questions as there are actual empirical articles on the subject”^1^; “Here's my challenge. Can anyone name one neuroscience study that provides insights into teaching that are likely to be useful before being confirmed by cognitive science?”.^2^

In this review, we indicate why neuroscience should have a role in education (and vice versa). We assess progress in the field of educational neuroscience and then outline major criticisms and future prospects.

## Why psychology is not enough

There is a long history of interaction between psychology and education (Bruer, 1997). In response to the emergence of educational neuroscience, it has been argued that the field of psychology is sufficient to inform education with a scientific understanding of learning processes. For example, Bowers claimed “psychology is the relevant discipline to improve educational outcomes for all children” (Bowers, 2016b, p. 633) and Bishop (2014) claimed that “the people who can be useful to teachers are psychologists.” This makes sense, because education concerns the effect of instruction on changes in behaviour. Psychology studies behaviour, while neuroscience is the study of brain mechanisms underlying behaviour.^3^

Yet there are risks in a pure psychology approach. Psychological theory infers hidden causal mechanisms to explain and predict observed behaviour. For example, it infers mechanisms such as ‘working memory’ and ‘attention’, which are then imbued with certain properties: working memory has a ‘limited capacity’; attention works like ‘a spotlight’ or enforces a ‘bottleneck’ on perception. What is the inspiration for these inferred mechanisms? Several heuristics are used, among them task analysis, reverse engineering and introspection. Historically, technological metaphors have also been influential, including hydraulic systems, steam engines and telephone exchanges. The symbolic/desktop computer has been particularly influential in modern cognitive psychology and is the origin of the concept of working memory. The symbolic computer employs general purpose processing components: a central processing unit, working memory, the hard drive and functions by abstracting information into a common format so that these general devices can process information of all different types. Research in computer science, artificial intelligence and machine learning has shown that such an engineering solution can create extremely powerful computational devices. However, this solution of domain‐general computation may not be one that is deliverable by the brain. The risk of a pure psychology approach, then, is that it can lead to erroneous theories based on inferred causal mechanisms that cannot be realised in real time by the brain (Mareschal et al., 2007).

Let us make this more concrete. Here are some education‐relevant questions that point to peculiar properties of learning: *Why am I liable to forget what the capital of Hungary is, but not to forget that I'm afraid of spiders? Why do I find I have learned things better after a good night's sleep? Why does my mind go blank when I'm stressed in an exam? I get 7/10 in a test – why am I delighted if I was expecting to get 5, demoralised if I was expecting to get 9? When I became a teenager, why did I sometimes do stupid, occasionally dangerous things just to impress my friends? Why can I learn a new language so much more easily when I'm 5 years of age than when I'm 50?* From a computer science perspective, one could imagine designing a machine learning system that did not have any of these peculiar properties. The machine learning system could store Capitals‐of‐European‐Countries and Animals‐I'm‐Scared‐Of as similar types of memories. It need not forget either. One could build the machine learning system without emotions like ‘stress’ or ‘anxiety’, emotions which on the face of it seem to detract from human learning performance. The system could be driven by absolute performance metrics so that a 7/10 is just a 7/10. Moreover, battery life permitting, one could build a system that did not need to sleep to achieve efficient learning. The answers to the above education‐relevant questions lie not in psychology, but in the particular way the human brain works, because of its particular biological and evolutionary origins. That is, there are aspects of learning that simply do not make sense except with respect to the brain.

There are several reasons to suspect that the set of inferred mechanisms that psychology currently uses is flawed because psychology has not paid sufficient attention to the constraints of neuroscience. First, as with the symbolic computer, proposed cognitive mechanisms tend to be general purpose: mechanisms such as working memory, long‐term memory, attention and cognitive control. When people train on a given task, their performance usually improves. Let's say a person trains on a task taken to involve one of these general mechanisms, such as working memory. One would then expect the benefits of training also to be observed across a wide range of other abilities assumed to utilise the same general mechanism. However, as a rule, such ‘far transfer’ is rarely observed; people tend to show improvements only on abilities similar to those on which they train (Sala & Gobet, 2017; Thorndike & Woodworth, 1901). This is not how one would expect a system to behave if it were based on domain‐general mechanisms. The implication is that the actual mechanisms used by the brain are less general than current cognitive theory supposes, and that it uses specific circuits for specific skills.

Second, there are behavioural observations that cannot be predicted from psychological theories, such as why the ability to learn should alter with age, or why forgetting should occur in particular ways for particular memories. Third, there are phenomena that seem surprising given current cognitive theory and which lead to ad hoc theoretical additions, such as the role of sleep in learning, or the effect of meditation on behavioural regulation (see later). Fourth, current psychological theories show a very poor match with the activation of neural structures, exhibiting numerous many‐to‐one relationships (where many cognitive processes are associated with the same brain region) and one‐to‐many relationships (where a single cognitive process is associated with multiple brain regions; Price & Friston, 2005). While the way that cognitive processes are implemented in the brain is of little relevance to educators per se, it becomes relevant if the cognitive primitives being proposed are wrong, because then teachers' expectations will be wrong. For example, if teachers believe attention is a single cognitive process of ‘focusing on one thing’, they may expect certain behaviours to develop together and be trainable together. However, the multiple brain areas involved in attention suggest that it is not one thing but a cluster of overlapping mechanisms, which involve, respectively orienting for action, top–down contributions to stimulus detection and sustained maintenance of task set (e.g. Petersen & Posner, 2012). These mechanisms may not develop in harness or be equally trainable.

These reservations about current cognitive theory are not principled limitations for psychology. They merely suggest psychology needs better theories. Theoretical advances will occur more quickly with closer ties to neuroscience, so that less time is spent considering possible ways the cognitive system could have worked, and more time considering how it actually works in the brain. In the meantime, for educators, psychological constructs such as working memory remain a double‐edged sword. They can be a useful summary of behavioural observations. A child with problems in ‘working memory’ will have problems keeping in mind a list of instructions to follow, a set of moves to make, or a sequence of numbers, and teachers can adjust classroom learning environments to mitigate these difficulties (Alloway & Alloway, 2014). Yet if teachers expect training working memory to yield general improvement across a diverse range of abilities, they would be wrong (Sala & Gobet, 2017; Simons et al., 2016).

This model of interaction between fields – of neuroscience with psychology, and then psychology with education – has been advanced by a number of researchers, from Bruer (1997) onwards (e.g. Howard‐Jones et al., 2016; Mareschal, Butterworth, & Tolmie, 2013; Varma et al., 2008). Crucially, it requires that evidence from neuroscience is used to modify psychological theory, rather than merely demonstrating how the brain ‘implements’ current cognitive theory – that is, using brain imaging to produce a list of brain regions that are activated during the operation of each putative cognitive process. Such a list offers little added value to educators.

**Figure 1:**
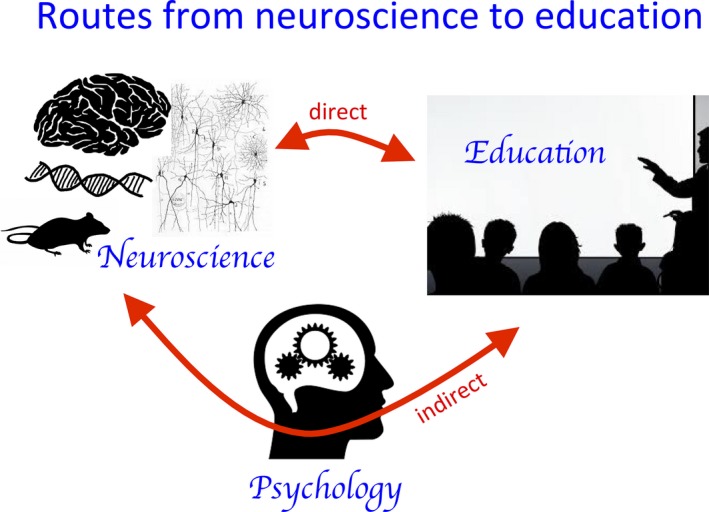
Two pathways linking neuroscience to education [Colour figure can be viewed at wileyonlinelibrary.com]

However, this indirect, two‐step route between neuroscience and education is not the only possibility. By virtue of the fact that the brain is a biological organ and therefore subject to metabolic constraints, there may be direct links. Factors such as energy supply, nutrition, response to stress hormones and environmental pollution can potentially influence brain function, including learning. Thus, while educational neuroscience predominantly places psychology at its centre, research on the impact of non‐psychological factors on educational outcomes, such as aerobic fitness, diet and air quality, also falls within its remit. These two pathways are shown in Figure 1.

## Challenges faced by educational neuroscience

Before we consider areas where educational neuroscience has made most progress, it is worth pointing out why the goal of the field is a very challenging one. First, the way the brain learns is complex. Second, learning is only one part of education. Third, society's goals for education are not necessarily clear. And fourth, even for psychology, successful translation from science to educational practice has proved difficult. We consider these points in more detail below.

While ‘learning’ is something that (hopefully) happens in the classroom, the term has multiple realisations in the brain. To illustrate, here is a thumbnail sketch that identifies eight interacting learning systems in the brain: (a) There is a system for memorising specific moments, which produces episodic or autobiographical memory. This is the hippocampus and the structures around it. This system can change its connections very quickly to record snapshots; (b) The brain learns associations between perceptual information and motor responses. It spots complex spatial and temporal patterns within this knowledge, so‐called ‘concepts’. This happens within the cortex, where changing connections takes seconds, minutes and hours; (c) Some associations are unconscious and involve the emotion (limbic) structures further inside the brain, associations between stimulus and response usually referred to as ‘classical conditioning’. These associations can form over seconds and minutes; (d) The brain learns to control content‐specific systems in posterior cortex so that they are activated in the appropriate contexts. Control involves the prefrontal cortex, which also interacts with limbic structures to integrate planning with emotion; (e) There is a reward‐based system that works out what we have to do to get what we want, to make nice things happen and avoid bad things happening, which operates over seconds and minutes; (f) There is a procedural learning system for learning activities that we perform frequently and often unconsciously, such as tying shoelaces, reading or driving a car. These automatic skills can take tens or hundreds of hours to learn through practice. The structures involved are the looping outer‐to‐inner circuits connecting the cortex through the basal ganglia to the thalamus and back again, and the cerebellum; (g) The brain can take advantage of its widespread circuits for perceiving and understanding other people, so that skills can be learned simply by observing other people, so‐called ‘modelling’; (h) The brain can take advantage of its widespread circuits for using language to construct new concepts and plans, so that skills can be learned through instruction.

In addition to these multiple systems, a broader principle operates: to make all processes automatic, so they occur quickly, smoothly and without need for cognitive effort or even awareness. Skills are progressively transferred to basal ganglia and cerebellar structures. The more knowledge/skills are used, the more they become automatic. By the same token, the less they are used, the more the skill or knowledge is likely to be lost. Forgetting happens at a different pace in different learning systems. All of these systems work in an integrated fashion; they respond differently over time and to regimes of training; and they can be differentially modulated by other factors, such as motivational and emotional state. In face of this complexity, understanding the implications of this constellation of mechanisms for the term ‘learning’ as construed by educators is a huge challenge.

**Figure 2:**
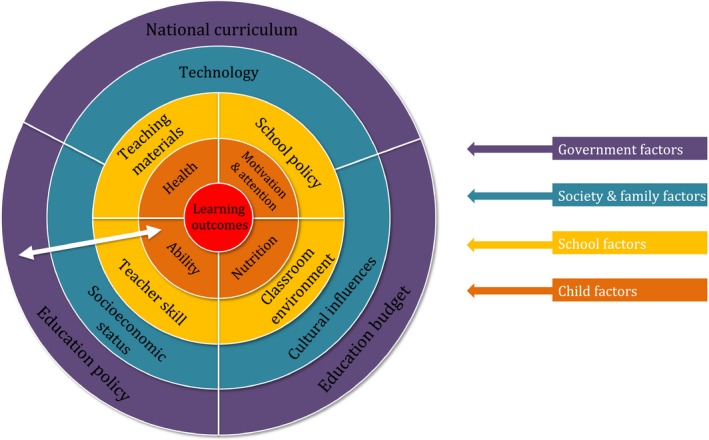
Proximal and distal factors that support and constrain change in learning outcomes, following the layered influences on behavioural change proposed by Michie et al. (2011), and the interactive relationships between an individual and his or her environment as proposed by Bronfenbrenner (1992). The white arrow reflects bidirectional influences between layers. The inclusion of ‘Technology’ at the level of Society and Family Factors illustrates children's use of digital media to engage with information outside of the classroom, but technology also contributes to the way information is presented in the classroom, shown here as ‘Teaching materials’ [Colour figure can be viewed at wileyonlinelibrary.com]

Even if educational neuroscience succeeds in this challenge, learning is only one part of education. Inspired by Bronfenbrenner's ecological systems theory (Bronfenbrenner, 1992), Figure 2 places learning outcomes at the heart of education, but illustrates the range of other factors, governmental, societal, institutional and child‐internal, which make up the broader picture. As Bronfenbrenner's writings demonstrate, the factors that influence a child's learning outcomes, which operate at vastly different degrees of proximity to the learning process, should be seen as an interactive, interconnected system. The goal of educational neuroscience is to improve educational outcomes, largely by changing the most proximal factors to learning outcomes shown Figure 2: ability, motivation and attention, health and nutrition. However, it should be borne in mind the range of barriers to change that may be encountered beyond optimising learning itself; behavioural change needs to be considered within the wider framework of implementation science (e.g. Michie, van Stralen, & West, 2011).

Policymakers seek to put in place the right structures to deliver the educational outcomes desired by society. This involves deciding on types of schools, types of educators, subjects; when children should begin and leave school; who needs what qualifications and who needs what resources; the types of examination and assessment; and so forth. These decisions are predicated on having a clear goal for the education system. With clear goals, a study of learning mechanisms can inform the best way to reach them. However, the goals are not always clear. In particular, it is not clear whether policy changes are intended to improve outcomes for all, moving up the performance level of the whole population – for example, raising the mean performance of one country above another in international league tables – or to alter the gaps between children – for example, a policy to ‘leave no child behind’. Factors that influence population means may differ from those that alter the shape of population distributions, as research into historical changes in intelligence levels has illustrated (e.g. Flynn, 2012). For example, in industrialised nations, access to technology and improved nutrition have both been hypothesised as potential driving factors for population‐level increases in IQ‐test performance, but while experience with technology may act to move everybody's performance up, improvements in nutrition likely work to decrease variability in the distribution, preferentially benefitting those from lower SES backgrounds (see Pietschnig & Voracek, 2015).

We know that from multiple different perspectives, increasing mean education level translates to positive outcomes for societies (Lindahl & Krueger, 2001; Lutz, Creso Cuaresma, & Sanderson, 2008; Viner et al., 2017). However, the notion of mean education level conflates educational excellence with educational equality; high mean performance could be achieved either by the work of a few excellent performers or by low educational inequality with few poor performers (Schmidt & Burroughs, 2016). Hence, which educational strategies most effectively and efficiently promote growth? Is it wiser for countries to maximise performance at the top of the distribution at the risk of increasing the dispersion of performance or to minimise variability by boosting performance at the bottom? One international study of mathematics achievement in 13–14 year olds found a negative correlation between the dispersion of test scores and median test score for that country (Freeman, Machin, & Viarengo, 2010). Similarly, the mean educational achievement of a country has been found to be positively associated with a smaller impact of family background on achievement (Hermann & Horn, 2011). These studies suggest that countries gain more in terms of educational outcome by decreasing educational inequality, and that the study of learning mechanisms should inform policymakers as regards the most efficient ways to promote educational equality.

Finally, even with a solid understanding of the science of learning, translation into classroom practice is difficult. With its much longer, 125‐year history of the study of learning, psychology still struggles to properly inform teaching practices. Techniques persist in the classroom even when there is a large body of evidence indicating a lack of effect (Roediger, 2013). For example, teachers still encourage students to highlight/underline text and reread text to enhance learning, despite evidence from psychology that neither is effective (Dunlosky, Rawson, Marsh, Nathan, & Willingham, 2013). It is not straightforward to translate an understanding of how learning occurs in the brain into ways to improve learning outcomes through different types of instruction nor is it straightforward to translate an understanding of the cause of learning impairments into appropriate interventions (Bowers, 2016a).

Part of the issue is to determine what teachers need to know about neuroscience or psychology theory. Teaching in the classroom is an interactive skill delivered in the moment, so it cannot be directly driven by theory (Howard‐Jones et al., in press). Indeed, Willingham (2018) has recently argued that teachers do not even need to know psychological theory, let alone neuroscience. Rather, teachers should be familiar with behavioural observations – developmental patterns and consistencies in children's cognition, motivation and emotion, equating to “understanding children”. In this view, training for teachers should comprise practice in applying principles in context. Therefore, while it is intuitive that scientific insights into learning mechanisms should help improve educational outcomes, translational sciences have many hurdles to clear.

## Progress in educational neuroscience

In this section, we consider a recent funding round for projects in educational neuroscience to illustrate which types of projects are being invested in. We then consider the main areas of established research findings, in areas of brain health, cognition and core skills, adolescence, executive function, social cognition, emotion and developmental disorders, as well as the quest for techniques that will yield general enhancement of cognition.

### Example projects

In the United Kingdom in 2014, the Education Endowment Foundation (a charity with the mission to improve the educational attainment of the poorest pupils in English schools) and the Wellcome Trust launched an “Education and Neuroscience scheme”. The goal was to provide funding for collaborative projects between educators and neuroscientists to develop evidence‐based interventions for use in the classroom (e.g. Howard‐Jones, 2014b). The six research projects the scheme funded, some of which are still ongoing, give a snapshot of the make‐up of current research initiatives in educational neuroscience.^4^ One project examined sleep patterns in adolescents. It trialled later school start times, along with a sleep education programme, to assess their impact on teenagers' educational achievement (Espie et al., 2016; Kelley, Lockley, Foster, & Kelley, 2015). Another project investigated the effect of medium to high cardiovascular activity on academic attainment, using brain imaging to investigate the mechanistic basis of any observed correlation (see Johansen‐Berg & Duzel, 2016).

The next project investigated the potential of inhibitory control training on primary age children's learning of mathematics and science, based on neuroscience evidence that conceptual learning in these domains requires suppression of pre‐existing beliefs, particularly for counterintuitive concepts (Mareschal, 2016) – for example, learning the world is round when it seems flat. The fourth project assessed the effectiveness of ‘spaced learning’, a particular regime of repeating a unit of work three times interspersed with alternative activities (Kelley & Whatson, 2013). The fifth project drew on the neuroscience of reward‐based learning, evaluating the effectiveness of uncertain reward on attainment in science (Howard‐Jones & Jay, 2016). The final project investigated the potential of a computer game to improve reading via developing phonological awareness through ‘rhyme analogy’ (Kyle, Kujala, Richardson, Lyytinen, & Goswami, 2013).

### Brain health

The first two projects illustrate the direct route from neuroscience to education. The direct route seeks to improve educational outcomes by enhancing the operation of the brain as a biological organ. This can be viewed as part of a broader approach of improving ‘brain health’ or of ‘brain optimisation’, concepts that extend beyond the school walls to the lifestyles of children and adults at home. Brain health targets physical fitness, diet, sleep and stress reduction through relaxation or meditation techniques and considers environmental factors such as air and noise pollution. By addressing these factors, the child should arrive in the classroom in the optimal state to learn. For example, restricting adolescent sleep time to just 5 or 6 hours a night for several nights has a marked impact on cognitive performance, most consistently on working memory ability (Jiang et al., 2011; Lo, Ong, Leong, Gooley, & Chee, 2016), even in the absence of subjective feelings of sleepiness. Conversely, increasing the regularity and intensity of physical exercise during the school day typically improves both academic performance and cognitive test scores (see Ruiz‐Ariza, Grao‐Cruces, Marques de Loureiro, & Martínez‐López, 2017). The role of relaxation and meditation practice on learning outcomes is also an area of active research. Meditation putatively serves not just to reduce stress but perhaps also to improve executive function skills and emotion regulation by helping children actively engage and direct their own attention. Although published intervention studies have not yet looked at the impact of meditation on academic performance, research indicates a positive impact on prosocial psychosocial attributes such as classroom behaviour and a decrease in reported and observed psychopathology in children (see Felver, Celis‐de Hoyos, Tezanos, & Singh, 2016).

Epidemiological studies have suggested that highly polluted environments are associated with delays in cognitive development as shown, for example, by a recent prospective study of 7–10‐year‐old children (*n* = 2,715) in Barcelona (Sunyer et al., 2015). Proposals have been advanced for the biological basis of this effect, such as translocation of inhaled ultrafine particles into the brain, with associated induction of a low‐level chronic neuroinflammation and oxidative stress, resulting in glial activation and white matter injury (Donaldson et al., 2005). Notably, in focusing on biology, the direct route makes use of one particular neuroscience method, animal models. It can be difficult to infer specific causal mechanisms from naturalistic data sets containing complex sets of correlations between environmental factors and educational outcomes. In animal models, a factor like air pollution can be randomly assigned and physiological changes can be observed in neurons that support learning. Establishing causal links between pollution and brain damage provides a much more compelling argument for stricter limits on pollution near schools. Without this kind of evidence, naturally existing correlations between pollution and poverty might make it difficult to offer policy recommendations.

### Cognition and core skills

The other four example projects illustrate the indirect route. They build on developmental cognitive neuroscience theories to propose and evaluate novel learning activities and their transfer to educational achievement. Respectively, the projects draw on theoretical work on concept formation and its relation to inhibitory control (that is, the capacity to voluntarily regulate strong or automatic behavioural responses), long‐term memory formation and its relation to long‐term potentiation, reward‐based learning and dopaminergic modulation of hippocampal learning, and brain sensitivity to print generated when children learn letter to speech sound correspondences at different levels of granularity.

One of the major strategies in the indirect route has been to use neuroscience evidence to help identify the core cognitive skills required for acquisition of domains such as literacy and numeracy, with the potential to identify the causes of deficits (for example, in one or more of the core skills) and training techniques that would target these skills. Work in numeracy has used imaging methods to understand the relationship between the learning and the respective core skills of the perception of number symbols (in fusiform gyrus in occipitotemporal lobes), representation of numerosity and manipulations of quantities (respectively, in intraparietal sulcus and the angular gyrus within the parietal lobe), spatial abilities (in the parietal lobe) and concepts, principles and procedures (involving prefrontal cortex; Butterworth & Varma, 2013). To illustrate the kinds of questions currently under consideration, a theoretical debate continues on whether the approximate non‐symbolic representations of number (e.g. dot arrays) form the basis for symbolic number processing (Merkley & Ansari, 2016), or whether more abstract representations are necessary to forge the connections between language, spatial representations and perceptual attention systems for tracking small numbers of objects. The identification of a set of core underlying systems has raised the possibility of training the parietal ‘approximate number systems’ for processing quantity (Budgen, DeWind, & Brannon, 2016), visuospatial working memory for counting and subitising (Menon, 2016) and spatial processing for using material such as maps, diagrams and graphs (Newcombe, 2016).

Research in other academic disciplines is less advanced. Science comprises a diversity of skills, including concept formation and reasoning. The recent focus has been on how the acquisition of scientific concepts can be integrated with prior intuitive concepts, for instance about the nature of the physical world (e.g. the Earth seems flat, heavy objects seem to fall faster than light ones) and on how intuitive reasoning can be extended to hypothesis testing (see Mareschal, 2016; Fugelsang & Mareschal, 2013). There is, relatively speaking, less topic‐specific educational neuroscience pertaining to topics such as history, geography, art and sports (though perhaps music is an exception, see e.g. Koelsch, 2012; Peretz & Zatorre, 2003,).

### Adolescence

There has been a significant amount of work in educational neuroscience into adolescence (see, e.g. Blakemore, 2018; Fuhrmann, Knoll, & Blakemore, 2015). Notably, the focus on adolescence was sparked by neuroscience evidence that the brain exhibits an extended developmental trajectory continuing through adolescence and into early adulthood (e.g. Dumontheil, 2016, for review). For example, although it was thought intelligence stabilised by 10 years of age, justifying educational selection according to ability at aged 11, Ramsden et al. (2011) demonstrated that an individual's IQ score, measured with standardised verbal and non‐verbal tests, can fluctuate across adolescence. Importantly, changes in brain structure closely mirrored the changes in IQ, indicating that fluctuating IQ scores were not simply the result of errors in test administration, design, coding or participant motivation, but represented real changes in ability. Moreover, when Knoll et al. (2016) compared training of relational reasoning against a control task of face discrimination in a sample of over 600 individuals from 11 to 33 years, they found that gains in relational reasoning were stronger in older adolescence and young adulthood than in early adolescence. Meanwhile, training on the perceptual task of face discrimination yielded no reliable gains in any age group. For the more complex task, learning ability was greater in the older groups.

The extended developmental trajectory in adolescence occurs unevenly across the brain, with temporal and frontal areas the last to mature. It has been argued that this aspect of brain development, possibly driven by pubertal hormonal changes (Piekarski, Boivin, & Wilbrecht, 2017), might offer insights into late developing skills in adolescence, such as (responsible) decision‐making and perspective taking (e.g. Mills, Goddings, Clasen, Giedd, & Blakemore, 2014). In addition, there may be specific changes linked to puberty that alter reward‐based processing in adolescence and vulnerability to peer influence, a particular characteristic of the teenage years (Crone & Dahl, 2012; Van Hoorn, Fuligni, Crone, & Galvan, 2016).

### Executive functions, social cognition and emotion

While branches of educational neuroscience address cognitive skills relevant to particular academic domains, there is also work that considers cognitive processes that impact all domains. Perhaps the most studied are ‘executive functions’, a set of related processes involving cognitive control and flexibility (Diamond, 2013). Executive function skills are strongly predictive of academic achievement, for example, predicting up to a third of the variance in maths and reading scores (Best, Miller, & Naglieri, 2011; Samuels, Tournaki, Blackman, & Zilinski, 2016). Cognitive neuroscience research continues to investigate how the improvement of executive functions with age is constrained by the development of relevant prefrontal brain regions (Kharitonova, Martin, Gabrieli, & Sheridan, 2013). This is relevant not only for the role of executive functions in subsequent academic achievement but also, at a younger age, for school readiness – how well a child can follow instructions, engage and take part in the learning environment of the classroom (Blair, 2016; De Haan, 2013). Executive function skills are potentially trainable (Diamond & Ling, 2016) and, importantly, are a remediable weakness in children raised in deprived home environments (Neville et al., 2013).

Educational neuroscience research on cognitive control also extends to the regulation of emotion. It is sometimes necessary for children to regulate emotions in educational contexts, and this is a skill that develops across early childhood (McRae, 2016). Here again, researchers have investigated how emotion regulation is associated with the development of a set of prefrontal regions involved in executive functions and their connections to limbic systems (Martin & Ochsner, 2016). Emotion has been considered in relation to specific academic domains, particularly with respect to maths anxiety (Beilock & Maloney, 2015; Chang & Beilock, 2016). However, emotion has a wider relevance to the social classroom environment and the relationships between students and between students and teachers (Immordino‐Yang & Gotlieb, 2018). Research into adolescence has focused on motivation and psychological well‐being, with heightened risk of mental health problems in the teenage years (Jones, 2013). Finally, work in educational neuroscience has begun characterising the development of the social brain (Blakemore, Kadosh, Sebastian, Grossman & Johnson, 2013). This includes mechanisms underlying abilities such as gaze processing, joint attention, face processing, action observation and reasoning about other people's mental states; for adolescence, one particularly important area is peer acceptance and rejection (Van Hoorn et al., 2016).

In contrast to work on executive functioning, where specific training interventions are common, research on social and emotional processing is not as advanced in translating insights from cognitive neuroscience into classroom implications, despite recognition of the important role of schools in pupils' social and emotional development (UK Department for Education, 2007).

### Developmental disorders

Bruer (2013) argued that the most likely payoff for educational neuroscience will be in the area of special education, addressing the needs of children suffering from developmental language disorder, dyslexia, dyscalculia, attention deficit and related executive function disorders, and social and emotional disorders. Much research effort in cognitive neuroscience has been dedicated to these areas (e.g. Rinehart, Bradshaw, & Enticott, 2017). The goal is to identify the underlying mechanistic causes of the deficit, be they in brain areas that are under developed, brain areas that are under‐activated, or brain areas that are poorly connected; and to use these data to inform cognitive theories. The search for mechanistic explanations contrasts with and complements research approaches in educational psychology that address issues of exclusion/inclusion of children with special educational needs (SEN) from mainstream education and the psychosocial perspective of families with children with disabilities (see, e.g. Woolfson, 2011). However, a mechanistic understanding of the cause of a developmental deficit does not necessarily provide a straightforward pathway to an intervention to remediate the deficit – particularly when therapeutic interventions may exploit compensatory strategies as much as seek to alleviate the mechanistic deficit (Bowers, 2016a). Indeed, our current understanding of the mechanistic basis of compensatory strategies lags behind that of causes of deficits themselves (Thomas et al., 2018). Insights into the causes of deficits must be transformed by pedagogical principles into interventions, which in turn must be evaluated for their effectiveness by behavioural trials in educational contexts (Howard‐Jones et al., 2016).

One interesting case study to watch is the emergence of a new mechanistic theory of developmental dyslexia along with new avenues for intervention. The ‘temporal sampling’ theory of dyslexia (Goswami, 2017), motivated largely by findings from electrophysiology, proposes that the functional deficit in dyslexia is a difficulty using the temporal structure of speech to scaffold the perception of syllables and sentence prosody. To complement experimental work on this question, it has been found that highlighting auditory rhythmic information in non‐speech and speech stimuli is as effective at improving phonological awareness in children with dyslexia as directly targeting phonology (Thompson, Leong, & Goswami, 2013). Interventions for children with dyslexia may soon, therefore, focus on music and rhythm rather than reading (Flaugnacco et al., 2015; Goswami, 2017).

To date, there are good examples of neuroscience contributing to the understanding of the causes of developmental deficits, such as atypical development of parietal systems supporting number processing in dyscalculia (Butterworth, Varma, & Laurillard, 2011) or of the attention and executive function networks in prefrontal cortex in attention deficit hyperactivity disorder (Shaw et al., 2013). Studies of the brain mechanisms of learning may also allow better prediction of who will benefit from different intervention techniques. This has been shown in the case of children with dyslexia in whom brain responses during a reading task predicted response to intervention when behavioural measures held no predictive value (Hoeft et al., 2011). Neuroscience has also had a wider and subtler impact on the debate surrounding the utility of defining specific categories of learning disability and allocating diagnoses to individual children according to these categories. Investigations at both brain level (Peters, Bulthé, Daniels, Op de Beeck, & De Smedt, 2018) and genetic level (e.g. Kovas, Haworth, Dale, & Plomin, 2007) suggest that, genetic syndromes aside, most education‐relevant developmental deficits lie on a continuum with variation in abilities in the mainstream population. Moreover, little evidence has accrued for special teaching techniques applicable to specific development disorders that differ from techniques used with mainstream children, that is, for an SEN‐specific pedagogy (Davis & Florian, 2004). Educational neuroscience, therefore, contributes to the debate on the pros and cons of diagnosis and labelling. For example, there are many symptoms in common across struggling learners diagnosed with different categories of disorder, while symptom variability is very high for children with the same diagnosis (e.g. Gathercole et al., 2016). Such insights are to be pitted against potential pragmatic benefits of allocating diagnoses in helping to attract resources for, and guide the choice of, effective interventions.

### The pursuit of techniques to produce general benefits for cognition

As we have seen, cognitive training tends to produce only ‘near transfer’, that is, performance improvements to the task trained on or similar tasks, rather than more broadly across cognition (Sala & Gobet, 2017). Nevertheless, there remains a desire within educational neuroscience to find elusive activities that bring more widespread benefits to cognition. There is enthusiastic engagement from teachers/public/media when such possibilities are raised, enthusiasm which is sometimes in advance of the actual science. This trend for seeking general benefits can also be found within the commercial sector in so‐called ‘brain training’ products – though to date, commercial products have not yet achieved the advertised far transfer (Simons et al., 2016).

Within educational neuroscience, a number of possibilities are currently under investigation, among them executive function training (Diamond & Ling, 2016), mindfulness training (see Felver et al., 2016), playing chess (Sala & Gobet, 2016), action video game playing (Bediou et al., 2018), learning a musical instrument or a second language (Moreno, Lee, Janus, & Bialystok, 2015), sleep (Sharman, Illingworth, & Harvey, in press) and aerobic fitness training (see Ruiz‐Ariza et al., 2017). The jury is still out on many of these activities. Research is rendered more difficult on the one hand by the frequent lack of random allocation of participants to conditions, allowing for the possibility of confounds (e.g. that in some populations, bilinguals might also have higher education or SES levels; that those who successfully learn musical instruments may be more intelligent or dedicated to practise; that those who persistently play action video games may tend to have faster sensorimotor responses); and on the other hand, by the challenge of designing intervention studies to achieve random allocation. One recent review of the cognitive benefits of action video game playing suggested that intervention studies with random allocation produced effect sizes of around a third of the size of those observed in correlational studies without random allocation (Altarelli, Green, & Bavelier, in press). This suggests both possible effects of the training, and also that there are pre‐existing differences between the sorts of young adults who frequently play action video games compared to those who do not. After promises of broad benefits, detailed research has sometimes revealed that transfer is not as far as first anticipated [e.g. for action video games, the main transfer is to selective visual and auditory attention (Altarelli et al., in press); working memory training improves performance on other working memory tasks but not other components of executive functioning, such as cognitive flexibility or inhibition (Diamond & Ling, 2016; Melby‐Lervåg, Redick, & Hulme, 2016)] In any event, for each of these putative generally beneficial activities, it is desirable for investigators to propose and evaluate the cognitive and brain structures that mediate the transfer from training task to other cognitive skills. The less plausible the underlying mechanistic basis for the transfer, the more critically the published evidence in favour of the transfer must be examined.

## Main criticisms of educational neuroscience

Educational neuroscience has received diverse critiques, challenges and sources of support. Some of these do not pertain to the field of science itself. For example, there is a great deal of research into so‐called *neuromyths*. Neuromyths are misconceptions about the brain and education among teachers and the public that are either not yet supported by the data or actively contradicted by existing science (e.g. Goswami, 2006; Howard‐Jones, 2014a; Macdonald, Germine, Anderson, Christodoulou, & McGrath, 2017); examples include large benefits of students adopting a ‘growth mindset’ (Sisk, Burgoyne, Sun, Butler, & Macnamara, 2018) or teachers matching the presentation of teaching materials to students' individual ‘learning styles’ (Rohrer & Pashler, 2012). While this is an important issue in the communication of science and for the interaction between the stakeholders within educational neuroscience,^5^ it is not core to the enterprise of understanding the actual neuroscience of education, nor are miscommunications of science unique to educational neuroscience. Then, there is research on the so‐called *seductive allure of neuroscience*, the finding that proposed new teaching techniques are more likely to be believed when accompanied by brain images (Farah & Hook, 2013; Weisberg, Keil, Goodstein, Rawson, & Gray, 2008). While this is an important issue of contextual framing in education research, again it does not concern the enterprise of educational neuroscience itself.

The substantive criticisms of the field to date come in three types: a priori arguments against the relevance of neuroscience to education, criticisms of the current practical operation of the field and doubts about the viability of neuroscience methods for diagnosis of disorders or prediction of individual differences.

Regarding the first type of criticism, some commentators have viewed neuroscience data as in principle not relevant to education. For example, Bowers (2016a) views education as solely concerning the effect of instruction on behavioural outcomes and does not view the underpinning neural mechanisms of learning as pertinent to this question. “My claim is that neuroscience is irrelevant to the design and evaluation of teaching” (Bowers, 2016b, p. 630). As we saw with Willingham's (2018) recommendations, this crit icism may also be levelled at psychology. A variation of this criticism is that (perhaps thus far) neuroscience has not led to new methods or insights that were not already suspected based on behavioural evidence, or not in need of verification using behavioural evidence. The measurement and assessment of behaviour, in this view, are solely the purview of psychology. This criticism is sometimes exaggerated by equating neuroscience with functional brain imaging (rather than understanding the discipline to be the investigation of neural mechanisms by a diverse set of methods, see footnote 3); if education is about behavioural change, it surely does not matter whether or not there are correlated changes in brain activity (see, e.g. Bishop, 2014). A more philosophical variety of the criticism is that educational neuroscience is inappropriately reductionist: to the extent education is a social phenomenon, social problems require social solutions, not reduction to neural mechanisms (e.g. Breckler, 2006; Lalancette & Campbell, 2012; for discussion). These criticisms broadly represent a resistance to interdisciplinary research. In the earlier section, ‘Why psychology is not enough', we indicated possible disadvantages to this resistance.

Regarding the second type, there have been practical criticisms about the way research is conducted and the pace of progress (e.g. Anderson & Della Sala, 2012). Much of the initial research in the field is likely to focus on understanding why the teaching methods that work *do* work, but there are expectations that neuroscience must quickly yield revolutionary teaching methods of large effect size (Thomas, 2013). Additionally, the methods required to collect neuroscience data, such as brain imaging, require controlled experimental situations. These are far from the context of naturalistic behaviour in the classroom and therefore of questionable validity. Furthermore, the neuroscience data themselves can be complex and hard to interpret. In terms of interdisciplinary dynamics, most educational neuroscientists advocate a dialogue between disciplines, but critics suggest that so far, it has constituted neuroscientists (patronisingly) lecturing teachers about what they should be doing in the classroom, with insufficient driving of the neuroscience research agenda by teachers (e.g. Turner, 2011). Where neuroscience has influenced educational policy and practice, it has been premature (Bruer, 1997). Broadly, these criticisms represent the growing pains of the field and areas where it must improve.

The last type of criticism has been in response to the proposed use of neuroscience data to predict developmental outcomes, such as dyslexia or autism, for example, using functional brain imaging or genetics. Neuroscience methods offer some advantages: they can distinguish different underlying causes of the same behaviour (Sheridan & McLaughlin, 2016), they can reveal processes not evident in behaviour (e.g. inhibition of task‐irrelevant responses), and when collected early, they can have predictive power for developmental outcomes. However, it is argued that neuroscience methods do not yet have the specificity or sensitivity to be useful screening methods, that their cost is too high, and that practicality much poorer compared to existing, simpler behavioural methods (e.g. Bishop, 2013, 2014). These are reasonable criticisms of the current state of the technology and evidence base but may not always be so. Neuroscientific measures have the potential to complement behavioural or environmental risk factors by being available long before a child starts school, for example, genetic tests at birth, or electrophysiological measures of language processing during infancy, may predict dyslexia risk (e.g. Guttorm, Leppänen, Hämäläinen, Eklund, & Lyytinen, 2009). Early predictions will maximise the available time for intervention and may be more effective by virtue of being implemented earlier in development, reducing risk when children start school.

## Future prospects and policy implications

We began by noting a comment by a leading educator that there seemed more papers about the potential of educational neuroscience than basic findings. It should now be apparent why this is. The ‘hype’ arises because it makes intuitive sense that insights into brain mechanisms of learning are likely to inform teaching in schools. The mismatch is because the basic science is hard, given the multiple learning systems in the brain and factors that influence their operation, and because translation into the classroom is also hard.

There is a ready model for how that translation may take place. As Roediger suggests, a translational educational science could be like medicine, ‘a field in which new discoveries from the lab in work with animals are tested in small‐scale studies with humans and then in clinical trials with larger numbers of people. If the therapeutic practices pass these tests, they are introduced into clinical practice (with results continuing to be monitored for such issues as side effects)’ – and in Roediger's view, it could yield the kinds of advances that have been witnessed in medicine over the last century (Roediger, 2013, p. 2).

The randomised control trial (RCT) is indeed being transferred from medicine into educational neuroscience: the six projects funded in the United Kingdom by the Wellcome Trust and the Educational Endowment Foundation in 2014 were based on this design. Moreover, a forthcoming consensus paper on methodologies to evaluate behavioural interventions for cognitive enhancement (Green et al., 2018) advocates their use, albeit adding the proviso that studies needed to be distinguished hierarchically into those establishing feasibility, mechanism, efficacy and effectiveness. The advantages and disadvantages of RCTs in education are currently being debated (e.g. Gorard, Huat See, & Siddiqui, 2017). For educational neuroscience, a possible downside is that new learning activities or techniques derived from RCTs will tend to be prescriptive in nature – to be delivered by teachers as designed by researchers. This risks undermining the autonomy of the teacher in the classroom as well as the teachers’ facility to adapt techniques to individual learners, which in turn could reduce uptake. Moreover, it is possible that the type of cognitive enhancements suggested by an understanding of neural learning mechanisms will individually tend to have small effect sizes on educational outcomes; many enhancements applied together may generate a large effect on children's educational outcomes; but in isolation, the effectiveness of each enhancement may be hard to establish in an RCT.

Going forward, educational neuroscience will need to resolve the inherent tension between those who view it as a basic science, a kind of developmental cognitive neuroscience relevant to education (e.g. Gabrieli, 2016) and those who view it as a necessarily translational field, as illustrated by the above‐mentioned educational neuroscience funding that was only targeted at intervention trials or by the books published by researchers in the field (*The learning brain: Lessons for education*, Blakemore & Frith, 2005; *Neuroscience for teachers: Applying research evidence from brain science*, Churches, Dommett, & Devonshire, 2017). Moreover, it will need to address the academic inertia that produces resistance to interdisciplinary research.

Educational neuroscience will also need to address new ethical issues raised by the field (Knowland, in press). Its goal is to enhance the cognitive and so‐called non‐cognitive abilities of children. In this regard, educators may be sanguine – that, after all, is the goal of education, and many variations on educational techniques are currently in play in classrooms across the globe. But from the perspective of psychology and neuroscience, the participants are children, and more stringent ethical principles apply than for adults. For example, ethical issues arise in the use of predictive measures of educational outcomes, which have potentially lifelong implications for the child but cannot be consented by the child. More broadly, educational neuroscience research has so far been largely restricted to developed countries. But in the developing world, educational outcomes may be more conditioned by social factors, even nutritional factors, and therefore, a broader understanding of social and political factors is required, rather than just cognitive neuroscience factors.

Finally, educational neuroscience as a field needs to decide how to respond to the (welcome) desire for policymakers to include neuroscience evidence in their policymaking. Early critiques of the field challenged premature influence of research, in that instance of work on sensitive periods in brain development on policymaking in early years education (Bruer, 1999). Policymakers are still keen to integrate cognitive neuroscience findings to make evidence‐informed decisions (e.g. in the early years: UK Parliamentary Select Committee enquiry into the Foundation years and UK Government's life chances strategy, 2016 [see Thomas, 2017];^6^ in university education: Willetts, 2018). One major roadblock here is the need to build up an evidence base that is sufficiently large and convergent to put scientific (and ideally consequent economic) weight behind policy initiatives. Certainly, in some areas of educational neuroscience, such as the long‐term detrimental effects of toxic stress in childhood, evidence is sufficiently persuasive to inform policy (Center on the Developing Child at Harvard University, 2017). More specific proposals for change may be slower in coming to fruition. For example, experts have recently called for the abolition of the requirement that in order to receive intervention, children with atypically poor language must demonstrate relatively good non‐verbal intelligence (see Bishop, 2017 for a summary of this debate). This call has resulted partly from evidence that the mechanisms of developmental language disorder are not different for children with and without average non‐verbal ability. Change in guidelines and policy that trickles down to practice in schools and clinics is often slow. Engagement with policymakers is both a future goal and a significant challenge for researchers in educational neuroscience.

**Key points**

- Educational neuroscience is an interdisciplinary research field that seeks to translate research findings on neural mechanisms of learning to educational practice and policy and to understand the effects of education on the brain.
- Neuroscience and education can interact directly, by virtue of considering the brain as a biological organ that needs to be in the optimal condition to learn (‘brain health’).
- Or it can interact indirectly, as neuroscience shapes psychological theory and psychology influences education.
- Policymakers remain keen to integrate cognitive neuroscience findings to make evidence‐informed decisions about education.

## Notes

1 Daniel Willingham, Professor of Psychology at the University of Virginia: https://twitter.com/dtwillingham/status/959439819557560320 (2 Feb, 2018; downloaded 10/4/18)

2 Dylan Wiliam, Emeritus Professor of Educational Assessment at UCL Institute of Education: https://twitter.com/dylanwiliam/status/959474461803237376 (2 Feb, 2018; downloaded 10/4/18)

3 For this article, we take neuroscience to be a research field with the goal of understanding the neural mechanisms underlying behaviour and to constitute a set of methods including brain imaging, brain anatomy, single‐cell recording, molecular methods, brain stimulation, animal models, genetics and computational modelling.

4
http://educationendowmentfoundation.org.uk/news/new-research-to-investigate-if-neuroscience-can-improve-teaching-and-learni/

5
http://www.educationalneuroscience.org.uk/resources/neuromyth-or-neurofact/

6
https://www.parliament.uk/business/committees/committees-a-z/commons-select/education-committee/inquiries/parliament-2015/foundation-years-and-the-uk-governments-life-chances-strategy-15-16/
